# A Randomized, Double-Blind, Placebo-Controlled Trial Assessing the Effects of Oral *Centella asiatica* Extract on Skin Aging-Related Parameters in Middle-Aged Korean Women

**DOI:** 10.3390/nu18101505

**Published:** 2026-05-08

**Authors:** Nayon Hur, Youngha Seo, Jaewoo Bae, Young Jun Kim, Eun Ji Kim, Yean Jung Choi

**Affiliations:** 1 3H LABS Research Institute, 3H LABS Co., Ltd., Goyang 10391, Republic of Korea; emily@3h-labs.com (N.H.); selena@3h-labs.com (Y.S.); jerry@3h-labs.com (J.B.); 2 Department of Food Regulatory Science, Korea University, Sejong 30019, Republic of Korea; yk46@korea.ac.kr; 3 Research Institute, SRE Service Co., Ltd., Chuncheon 24232, Republic of Korea; ejkim@sres.co.kr; 4 Department of Food and Nutrition, Sahmyook University, Seoul 01795, Republic of Korea; 5 Institute of Nutritional Physiology and Molecular Nutrition, Sahmyook University, Seoul 01795, Republic of Korea

**Keywords:** nutricosmetics, *Centella asiatica*, randomized controlled trial, skin aging, TEWL, skin elasticity

## Abstract

Background: *Centella asiatica* has been widely recognized for its dermatological benefits; however, clinical evidence supporting the efficacy of oral supplementation for improving skin aging parameters remains limited. This randomized, double-blind, placebo-controlled study evaluated the effects of oral *Centella asiatica* extract on skin wrinkles and related skin parameters in middle-aged women. Methods: A total of 112 participants were randomized to receive either *Centella asiatica* extract (200 mg/day) or placebo for 12 weeks. Skin wrinkle parameters were quantitatively assessed using a three-dimensional skin imaging system (PRIMOS^®^). Skin hydration, transepidermal water loss (TEWL), elasticity, and skin color (brightness and redness) were additionally measured using validated non-invasive instruments. Efficacy analyses were performed in the per-protocol population. Results: After 12 weeks of supplementation, the *Centella asiatica* extract group demonstrated significant improvements in multiple wrinkle parameters compared with the baseline. Average wrinkle depth decreased by 11.1%, and the mean depth of the largest wrinkle decreased by 14.4%. Maximum wrinkle depth and total wrinkle volume were reduced by 13.3% and 13.7%, respectively, while surface roughness (Ra) decreased by 10.4%. In contrast, the placebo group showed minimal or inconsistent changes. Epidermal hydration at the cheek site significantly increased, while transepidermal water loss decreased, indicating improved skin barrier function. However, the magnitude of changes in epidermal hydration (2.7%), skin elasticity (R2; 0.7%), and skin brightness (L*; 0.7%) were relatively small. Skin elasticity and skin brightness showed statistically significant differences compared with the placebo group (*p* < 0.05), but these changes should be interpreted as modest improvements. No serious adverse events were reported, and all hematological and biochemical safety markers remained within normal reference ranges during the 12-week intervention period. Conclusions: Oral supplementation with *Centella asiatica* extract for 12 weeks was associated with improvements in wrinkle-related parameters and TEWL, while changes in skin hydration, elasticity, and brightness were modest and of limited magnitude. These findings suggest a potential role for short-term supplementation; however, further studies are required to confirm long-term efficacy and broader applicability.

## 1. Introduction

Skin aging is a multifactorial biological process characterized by progressive alterations in skin structure, function, and appearance, including wrinkle formation, reduced hydration, impaired barrier integrity, and loss of elasticity [1,2]. These changes are driven by intrinsic aging as well as extrinsic factors, particularly ultraviolet (UV) exposure, which promotes oxidative stress, chronic low-grade inflammation, and degradation of extracellular matrix components such as collagen and elastin [3,4,5]. Impairment of the epidermal barrier, reflected by increased transepidermal water loss (TEWL), further exacerbates skin dryness and vulnerability to environmental stressors [6,7]. Consequently, maintaining skin health has become an important target not only in dermatology but also in nutrition and preventive health research [8,9].

Growing evidence indicates that nutrition plays a critical role in skin homeostasis and aging [10,11]. Dietary intake of bioactive compounds through foods or dietary supplements has been shown to influence skin hydration, elasticity, barrier function, and overall appearance by modulating antioxidant defense systems, inflammatory pathways, and lipid metabolism in the skin [9,12,13]. Unlike topical applications, oral nutritional interventions provide systemic support and may affect both superficial and deeper skin layers [10]. Accordingly, well-designed randomized controlled trials evaluating oral supplementation have become essential to substantiate health claims related to skin aging and skin health maintenance [14,15].

*Centella asiatica* (L.) Urb. is a medicinal plant traditionally used in Asian and Ayurvedic medicine for wound healing and various skin disorders [16,17,18]. Its major bioactive constituents include triterpenoid saponins such as asiaticoside, madecassoside, asiatic acid, and madecassic acid [19,20,21]. Preclinical studies have demonstrated that these compounds exhibit potent antioxidant and anti-inflammatory activities, promote fibroblast proliferation, enhance collagen synthesis, and protect extracellular matrix integrity [16,17,19,22,23,24]. In addition, *Centella asiatica*-derived triterpenoids have been reported to support epidermal barrier function and improve skin hydration through regulation of lipid organization and inflammatory mediators [25,26]. In line with these findings, our recent in vivo study demonstrated that oral administration of *Centella asiatica* extract (InnerCica^™^) significantly attenuated UVB-induced skin photoaging in hairless mice by enhancing antioxidant defense, suppressing inflammatory responses, and preserving extracellular matrix integrity [27]. InnerCica^™^ is a standardized extract derived from *Centella asiatica*, containing triterpenoid constituents such as asiaticoside, madecassoside, asiatic acid, and madecassic acid, which are known to contribute to its biological activity.

Despite these promising mechanistic findings, clinical evidence supporting the efficacy of orally administered *Centella asiatica* for skin health remains limited [28,29]. While *Centella asiatica* has been extensively studied in topical formulations, randomized controlled trials evaluating oral supplementation remain limited, and existing human intervention studies have often been constrained by small sample sizes, short intervention periods, or subjective outcome measures [25,30,31,32]. Moreover, there is a relative lack of high-quality trials employing objective, non-invasive skin measurement techniques to comprehensively assess wrinkles, hydration, barrier function, and elasticity following oral intake of *Centella asiatica* extract [33,34]. To date, no sufficiently powered randomized controlled trial has comprehensively evaluated the effects of orally administered *Centella asiatica* extract on multiple objective skin aging-related endpoints, including wrinkle morphology, hydration, barrier function, and elasticity.

Therefore, the present study aimed to investigate the effects of oral supplementation with *Centella asiatica* extract on multiple skin health parameters in middle-aged women. Using a 12-week randomized, double-blind, placebo-controlled design, we evaluated changes in skin wrinkles, hydration, transepidermal water loss, elasticity, and skin color (brightness and redness) using validated, quantitative, non-invasive instruments, while also assessing the safety and tolerability of the intervention through adverse event (AE) monitoring and clinical laboratory parameters. By providing controlled clinical data, this study seeks to clarify the potential role of *Centella asiatica* extract in supporting skin aging-related parameters within a defined study population.

## 2. Materials and Methods

### 2.1. Ethics Statement

This clinical trial was conducted in accordance with the principles of the Declaration of Helsinki and Korean Good Clinical Practice (KGCP) guidelines. All participants were provided with detailed information regarding the study objectives, procedures, potential risks, and benefits, and written informed consent was obtained from each participant. Participant confidentiality was strictly maintained throughout the study, and all data were anonymized prior to analysis. The study protocol (Protocol No. DEF-FFPIT053(1)-25003) was reviewed and approved by the Institutional Review Board (IRB) of the Dermapro Ltd. Skin Clinical Research Institute (approval number: 1-220777-A-N-01-DICN25001).

This study was registered with the Clinical Research Information Service (CRIS) of the Republic of Korea (http://cris.nih.go.kr, CRIS number: KCT0011582; registered on 6 February 2026). The study was conducted in accordance with the Consolidated Standards of Reporting Trials (CONSORT) guidelines for randomized controlled trials.

### 2.2. Study Participants

A total of 112 adults participated in this study. The inclusion criteria were as follows: (1) female participants aged 30 to 60 years with visible facial wrinkles in the facial area; (2) subjects with facial (cheek) skin hydration ≤48, as measured by a Corneometer^®^ (Courage & Khazaka Electronic GmbH, Cologne, Germany); (3) subjects with a wrinkle grade of ≥3 in the periocular (crow’s feet) area as assessed by a trained evaluator under standardized lighting conditions using a validated 10-point scale (0–9); (4) generally healthy subjects without acute or chronic systemic diseases, including skin disorders; (5) subjects who voluntarily provided written informed consent; and (6) subjects who were able to comply with all scheduled visits and study procedures. The exclusion criteria were as follows: (1) pregnancy, breastfeeding, or intention to become pregnant; (2) history of irritation or allergic reaction to cosmetics, pharmaceuticals, or foods related to the investigational product; (3) consumption of functional foods or dietary supplements containing skin-related active ingredients within 3 months prior to screening; (4) use of topical corticosteroids or systemic retinoids, corticosteroids, or immunosuppressive agents within 6 months prior to study initiation; (5) receipt of or plans to receive dermatological or cosmetic procedures (e.g., skin peeling, botulinum toxin injection, or other treatments) on the target area within 3 months prior to study initiation; (6) participation in another clinical trial within the previous 3 months; (7) presence of diseases that could affect the study outcomes, including cardiovascular, renal, hepatic, thyroid, cerebrovascular, gallbladder, and gastrointestinal diseases, or gout; (8) skin diseases (e.g., atopic dermatitis) on the target area; (9) chronic diseases such as asthma, diabetes mellitus, or hypertension; (10) psychiatric disorders, including depression, schizophrenia, alcohol use disorder, or drug dependence; (11) use of anti-obesity drugs, oral contraceptives, hormonal agents, or diuretics; (12) excessive alcohol consumption (≥40 g/day for men or ≥20 g/day for women); (13) sensitive or hypersensitive skin; (14) abnormal skin conditions on the target area, such as nevi, acne, erythema, or telangiectasia; or (15) any condition judged by the principal investigator to make participation unsuitable.

### 2.3. Study Design and Randomization

This study was conducted as a 12-week, randomized, double-blind, placebo-controlled, parallel-group human intervention trial designed to evaluate the effects of oral *Centella asiatica* extract supplementation on skin health parameters. This study was conducted at Dermapro Ltd. (Seoul, Republic of Korea) from January 2025 to May 2025. Following a screening period of up to three weeks (Visit 0), participants who satisfied all prespecified inclusion and exclusion criteria were enrolled and underwent baseline assessments. A total of 112 eligible participants were randomly assigned in a 1:1 ratio to either the *Centella asiatica* extract group (*n* = 56) or the placebo group (*n* = 56) using a computer-generated randomization schedule produced with the randomization function of SPSS version 20 (IBM Corp., Armonk, NY, USA) prior to study initiation. Both participants and investigators, including outcome assessors, were blinded to group assignments throughout the study period until completion of data analysis. Participants attended four study visits (Visit 1: week −3; Visit 2: week 0; Visit 3: week 6; and Visit 4: week 12). Baseline measurements were performed at week 0, and follow-up assessments were scheduled for 6-week intervals thereafter. Lifestyle-related questionnaires and dietary assessments were conducted at baseline and at the end of intervention. Information on outdoor exposure, sleep duration, facial cleansing habits, smoking and alcohol consumption was collected using standardized questionnaires. Dietary intake was assessed using 3-day dietary records collected prior to each visit, at baseline, and at week 12. Changes in nutrient and dietary intake were analyzed using CAN-Pro version 6.0 (Korean Nutrition Society, Seoul, Republic of Korea). Physical activity was recorded at baseline and at the end of the study. Participants were instructed to maintain their usual dietary habits, lifestyle patterns, and physical activity levels throughout the study period.

### 2.4. Study Product and Interventions

The study products evaluated in this trial were provided by 3H LABS Co., Ltd. (Goyang, Republic of Korea). The study product was prepared according to a previously described method [27]. Briefly, dried *C. asiatica* leaves were extracted twice with 70% (*v*/*v*) ethanol for 6 h at 80 °C, filtered, and concentrated. The concentrate was then spray-dried to obtain a powdered extract. The standardized *Centella asiatica* extract (InnerCica^™^) contained asiaticoside (70 mg/g) as the primary quality-controlled marker compound. Other triterpenoid constituents (e.g., madecassoside, asiatic acid, and madecassic acid) are known to be present in *Centella asiatica* extracts; however, their quantitative levels were not specified as part of the product’s quality-control criteria in this study. Participants assigned to the intervention group received 200 mg/day of the extract, administered orally as one tablet once daily, taken 30 min after a meal to improve gastrointestinal tolerability and to facilitate the absorption of lipophilic triterpenoid constituents (e.g., asiaticoside and madecassoside), whose bioavailability may be enhanced by postprandial lipid-mediated solubilization and intestinal uptake [35,36,37,38], for 12 weeks. The placebo group received an identically appearing placebo tablet formulated without the active ingredient, including microcrystalline cellulose and other food-grade additives without active components expected to influence skin-related outcomes. Both the test product and placebo were indistinguishable in appearance, taste, and packaging to maintain blinding.

### 2.5. Efficacy Outcome Measures

The prespecified primary endpoint was the change in average wrinkle depth (PRIMOS^®^) at week 12. This endpoint was selected a priori and used as the basis for sample size calculation. Secondary endpoints included other wrinkle-related parameters (e.g., maximum wrinkle depth, total wrinkle volume), epidermal hydration at the cheek site, and transepidermal water loss (TEWL). Exploratory endpoints included measurements at additional anatomical sites, skin elasticity, and pigmentation-related parameters (e.g., skin brightness and redness). All outcomes were assessed at baseline, week 6, and week 12 under controlled environmental conditions of temperature and humidity. Outcomes were analyzed as changes from baseline at each follow-up time point using validated, quantitative, non-invasive measurement instruments. Skin wrinkle parameters were evaluated using a three-dimensional optical skin imaging system (PRIMOS^®^ CR, Canfield, NJ, USA). Parameters analyzed included average wrinkle depth, mean depth of the biggest wrinkle, maximum wrinkle depth, total wrinkle volume, and surface roughness (Ra). Measurements were conducted on the facial cheek area following standardized positioning and calibration procedures. Epidermal hydration was assessed using a Corneometer^®^ (Courage & Khazaka, Cologne, Germany), which measures the capacitance of the stratum corneum. Dermal hydration was measured using a Moisturemeter^®^ D (Delfin Technologies, Kuopio, Finland). Measurements were performed at multiple anatomical sites; primary analyses focused on the cheek region. TEWL was measured using a Tewameter^®^ TM300 (Courage & Khazaka, Cologne, Germany) as an indicator of skin barrier function. Measurements were obtained under standardized environmental conditions after adequate acclimatization of participants. Skin elasticity was assessed using a Cutometer^®^ MPA580 (Courage & Khazaka, Cologne, Germany). The R2 parameter (Ua/Uf), representing gross elasticity, was selected as the primary elasticity index. Skin color parameters were evaluated using a Spectrophotometer^®^ CM-2500d (Konica Minolta, Tokyo, Japan). Values were recorded in the CIELAB color space, with skin brightness (L*) and redness (a*) used for analysis.

### 2.6. Safety Assessment

Safety was systematically assessed throughout the study period by monitoring AEs. All AEs were recorded, assessed for severity, and evaluated for their potential relationship to the study product. Blood samples were collected after an overnight fast of at least 8 h at screening (3 weeks prior to product administration) and after 12 weeks of supplementation. Laboratory analyses were conducted at a certified clinical laboratory and included measurements of glucose, aspartate aminotransferase (AST), alanine aminotransferase (ALT), γ-glutamyl transferase (γ-GTP), total cholesterol, triglycerides, high-density lipoprotein (HDL) cholesterol, low-density lipoprotein (LDL) cholesterol, and creatinine, as well as complete blood count parameters, including white blood cells (WBCs), red blood cells (RBCs), hemoglobin, hematocrit, neutrophils, lymphocytes, monocytes, and eosinophils. Vital signs and physical examinations were also performed throughout the study period.

### 2.7. Statistical Analysis

This study aimed to evaluate the impact of 12 weeks of *Centella asiatica* extract supplementation on skin health compared with a placebo. Sample size was calculated based on previously published randomized controlled trials evaluating wrinkle-related outcomes using objective skin imaging systems. Change in average wrinkle depth was defined as the primary outcome parameter for sample size estimation. Using a one-sided significance level of 5% and a statistical power of 95%, the minimum required sample size was estimated to be 48 participants per group. Considering an anticipated dropout rate of 20%, a total of 114 participants were planned for randomization. All statistical analyses were performed using SPSS (IBM Corp., Armonk, NY, USA). Continuous variables are presented as mean ± standard deviation (SD), and categorical variables are expressed as frequencies and percentages. The primary efficacy analysis was conducted in the per-protocol (PP) set, with additional analyses performed in the intention-to-treat (ITT) set for sensitivity assessment. Within-group changes from baseline to week 12 were analyzed using the paired *t*-test or the Wilcoxon signed-rank test. Between-group comparison of change values was evaluated using the independent *t*-test or Mann–Whitney U test. Categorical variables, including AEs and questionnaire responses, were compared between groups using the chi-square test or Fisher’s exact test, as appropriate. All statistical tests were two-sided, and a *p*-value < 0.05 was considered statistically significant. The primary endpoint (change in average wrinkle depth at week 12) was tested without adjustment for multiplicity. Analyses of secondary and exploratory endpoints were not adjusted for multiple comparisons and should be interpreted as supportive and exploratory.

## 3. Results

### 3.1. Demographic Characteristics of the Participants

**Figure 1:**
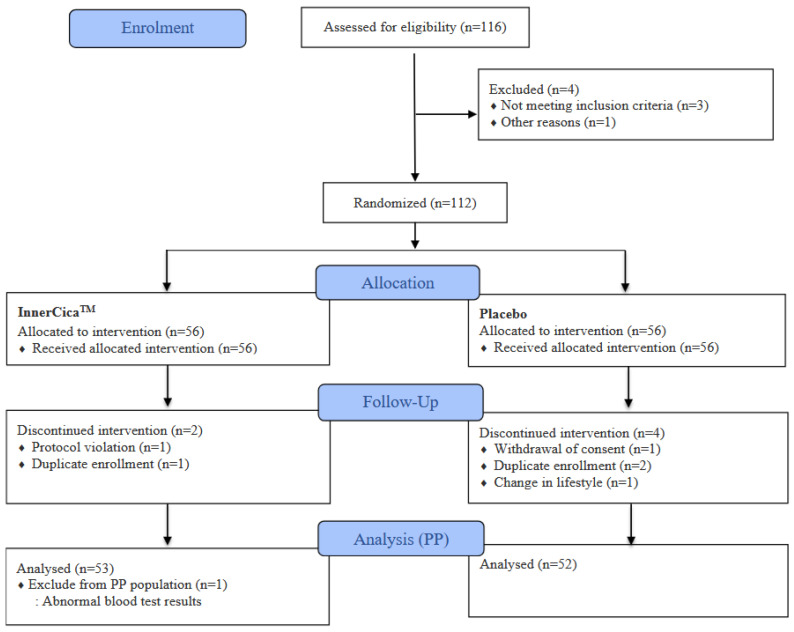
Study design and participant flow diagram. A total of 116 individuals were screened for eligibility, and 112 participants were randomized to receive either *Centella asiatica* extract (InnerCica^™^, *n* = 56) or placebo (*n* = 56). During the intervention period, six participants discontinued the study, and one participant was excluded from the per-protocol population due to abnormal blood test results. Finally, 53 participants in the extract group and 52 participants in the placebo group were included in the PP analysis.

A total of 116 participants were screened for eligibility, and 4 were excluded (3 because of failure to meet the inclusion criteria and 1 because of inability to obtain instrument measurements). The remaining 112 participants were randomized to receive either *Centella asiatica* extract (*n* = 56) or placebo (*n* = 56) in a 12-week randomized, double-blind, placebo-controlled, parallel-group trial (Figure 1). During the intervention period, six participants discontinued the study (extract group, *n* = 2; placebo group, *n* = 4) due to withdrawal of consent, duplicate enrollment, lifestyle changes, or protocol violation. In addition, one participant in the extract group was excluded from the per-protocol (PP) analysis because of abnormal blood test results. Consequently, 105 participants were included in the PP analysis, comprising 53 participants in the extract group and 52 participants in the placebo group. Participant allocation, follow-up, and analysis according to CONSORT guidelines are illustrated in Figure 1. A total of 112 participants were included in the intention-to-treat (ITT) population. The mean age in the ITT population was 56.45 ± 4.46 years in the extract group and 52.59 ± 5.06 years in the placebo group, with no significant difference between groups (*p* = 0.207). In the per-protocol (PP) population, the mean age was 51.51 ± 4.39 years in the extract group and 52.73 ± 5.18 years in the placebo group, which was also not significantly different between the groups (*p* = 0.195). Baseline self-evaluated skin parameters in the ITT population are summarized in Table 1, including skin type, skin hydration, sebum level, surface roughness, skin thickness, and skin sensitivity. No statistically significant differences were observed between the groups for any baseline skin parameters, indicating that the two groups were comparable before the intervention. Furthermore, body composition parameters in the PP population, including weight, body fat mass, body mass index (BMI), body fat percentage, and waist–hip ratio, are presented in Supplementary Table S1. These variables also showed no significant differences between the extract and placebo groups at baseline or after the intervention, confirming the comparability of the study groups. Compliance with product intake was high throughout the intervention period and exceeded 94% in both groups, with no significant difference between groups. These findings indicate good adherence to the study protocol.

### 3.2. Dietary Intake and Physical Activity

Lifestyle habits, dietary intake, and physical activity levels were evaluated to determine whether differences in daily lifestyle factors could influence the study outcomes. Analysis of lifestyle habits and dietary records indicated no significant differences between the *Centella asiatica* extract and placebo groups throughout the intervention period. Nutrient intake assessed from the dietary records also showed no statistically significant differences between the groups. These findings indicate that dietary intake and physical activity were comparable between groups and were unlikely to have materially influenced the study outcomes. Detailed results of lifestyle habits and nutrient intake are presented in Supplementary Tables S2–S4.

### 3.3. Efficacy Outcomes

#### 3.3.1. Effects on Periorbital Wrinkles

**Figure 2:**
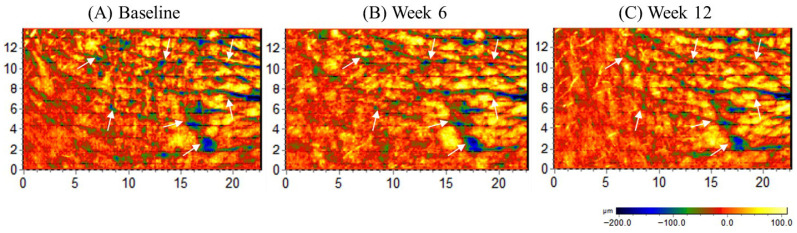
Representative improvement of periorbital wrinkles following oral supplementation with *Centella asiatica* extract. Representative three-dimensional skin surface images of the periorbital region obtained at (**A**) baseline, (**B**) Week 6, and (**C**) Week 12 using a PRIMOS^®^ three-dimensional skin imaging system. The pseudo-color scale represents wrinkle depth (µm), with cooler colors (blue–black) indicating deeper wrinkle regions and warmer colors (yellow–red) indicating shallower skin surface areas. Representative images from a participant in the *Centella asiatica* extract group illustrate a visible reduction in wrinkle depth and surface roughness over the 12-week supplementation period. Arrows indicate representative regions demonstrating reductions in wrinkle depth and surface irregularity following supplementation. Image size: 14 mm (H) × 22 mm (W). Surface topography is represented by a color-coded scale ranging from −200 μm (depressed regions, black/blue) to +100 μm (elevated regions, yellow).

Skin wrinkle parameters were quantitatively evaluated using a three-dimensional skin imaging system (PRIMOS^®^), and the results are summarized in Table 2. In the *Centella asiatica* extract group, several clinically relevant wrinkle parameters showed significant improvement compared with baseline. At week 12, the reduction in average wrinkle depth from baseline was significantly greater in the extract group than in the placebo group (−5.87 ± 7.82 μm vs. −1.96 ± 6.09 μm; *p* = 0.018). The average depth of wrinkles decreased from 53.02 ± 12.06 μm at baseline to 47.15 ± 8.29 μm at week 12, representing an approximately 11.1% reduction (*p* < 0.001), whereas the placebo group showed only a modest reduction (56.06 ± 11.53 μm to 54.10 ± 11.12 μm). Similarly, the reduction in the mean depth of the biggest wrinkle at week 12 was significantly greater in the extract group than in the placebo group (−10.36 ± 16.08 μm vs. −3.04 ± 12.58 μm; *p* = 0.011). The mean depth decreased from 72.19 ± 22.06 μm to 61.83 ± 15.50 μm (approximately 14.4% reduction; *p* < 0.001), whereas no significant change was observed in the placebo group. The maximum wrinkle depth decreased from 227.85 ± 91.99 μm to 197.57 ± 68.21 μm in the extract group (approximately 13.3% reduction; *p* < 0.001), while the placebo group showed smaller change (246.08 ± 92.97 μm to 231.65 ± 91.40 μm). Although the reduction tended to be greater in the extract group (−30.28 ± 67.14 μm vs. −14.43 ± 57.88 μm), the between-group difference did not reach statistical significance. Total wrinkle volume decreased from 2.27 ± 0.70 mm^3^ to 1.96 ± 0.50 mm^3^ in the extract group (approximately 13.7% reduction; *p* < 0.001), whereas the placebo group showed minimal change (2.36 ± 0.66 mm^3^ to 2.26 ± 0.63 mm^3^). The reduction was significantly greater in the extract group than in the placebo group (−0.30 ± 0.38 mm^3^ vs. −0.10 ± 0.33 mm^3^; *p* = 0.004). Surface texture parameters also improved following supplementation. Surface roughness (Ra) decreased from 24.60 ± 4.90 μm to 22.04 ± 3.54 μm (approximately 10.4% reduction; *p* < 0.001) in the extract group, whereas the placebo group showed a smaller decrease (25.83 ± 4.79 μm to 24.77 ± 4.62 μm). The reduction was significantly greater in the extract group (−2.57 ± 3.12 μm vs. −1.06 ± 2.84 μm; *p* = 0.023). Additional PRIMOS-derived parameters showed a similar pattern. The maximum height of the surface profile (Ry) decreased from 436.57 ± 117.28 μm to 396.15 ± 96.37 μm (*p* = 0.002), suggesting reduced surface irregularity. Overall, these findings demonstrate that oral supplementation with *Centella asiatica* extract was associated with statistically significant improvements in the prespecified primary endpoint (average wrinkle depth) after 12 weeks compared with placebo, while additional improvements observed in other wrinkle-related parameters were also statistically significant and should be interpreted as supportive findings. Representative three-dimensional PRIMOS images of the periorbital region before and after supplementation are presented in Figure 2, illustrating changes in wrinkle depth and surface roughness.

**Figure 3:**
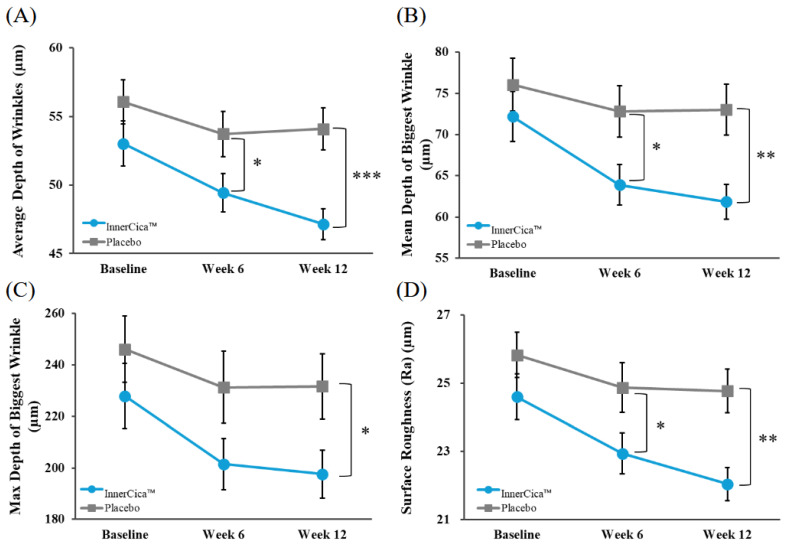
Changes in periorbital wrinkle parameters following oral supplementation with *Centella asiatica* extract (InnerCica^™^). Changes in key wrinkle parameters assessed using a PRIMOS^®^ three-dimensional skin imaging system at baseline, Week 6, and Week 12. The evaluated parameters include (**A**) average wrinkle depth, (**B**) mean depth of the biggest wrinkle, (**C**) total wrinkle volume, and (**D**) surface roughness (Ra). Values are presented as mean ± SD (per-protocol analysis). * *p* < 0.05, ** *p* < 0.01, *** *p* < 0.001 vs. placebo group.

In addition, the temporal trends and between-group differences in key wrinkle parameters are summarized in Figure 3. As shown, the *Centella asiatica* extract group exhibited a consistent and progressive reduction in wrinkle-related parameters over the 12-week intervention period, whereas the placebo group showed only modest changes. Notably, parameters such as average wrinkle depth, mean depth of the largest wrinkle, total wrinkle volume, and surface roughness demonstrated clearer separation between groups over time. In contrast, parameters including total wrinkle area and total wrinkle length showed minimal changes.

#### 3.3.2. Effects on Skin Hydration

Epidermal hydration was evaluated using a Corneometer^®^, and the results are presented in Table 3. In the *Centella asiatica* extract group, cheek skin hydration showed a significant increase over time, rising from 44.83 ± 4.73 A.U. at baseline to 46.89 ± 4.76 A.U. at week 6 (*p* < 0.001) and 48.09 ± 5.08 A.U. at week 12 (*p* < 0.001), corresponding to an approximately modest increase from baseline. In contrast, the placebo group exhibited a smaller increase from 44.10 ± 3.75 A.U. to 45.45 ± 4.17 A.U. over the same period. The between-group difference in change from baseline was significant at both week 6 (*p* = 0.008) and week 12 (*p* < 0.001). At other anatomical sites, changes in epidermal hydration were less pronounced and not consistently different between groups. Forearm hydration demonstrated a slight increase in both groups, with the extract group rising from 37.88 ± 7.35 A.U. to 39.28 ± 6.12 A.U. at week 12 (*p* = 0.001), while no significant between-group difference was observed. Similarly, dorsal hand hydration exhibited modest increases in both groups without significant differences.

Dermal hydration was assessed using a Moisturemeter^®^. In the *Centella asiatica* extract group, cheek dermal hydration increased significantly from 36.10 ± 2.64 A.U. at baseline to 37.08 ± 2.48 A.U. at week 12 (*p* < 0.001), whereas no significant change was observed in the placebo group (36.36 ± 3.23 A.U. to 36.32 ± 3.27 A.U.). The between-group difference in changes from baseline was significant at week 12 (*p* < 0.001). At the dorsal hand site, dermal hydration also showed a statistically significant increase in the extract group (38.31 ± 3.23 A.U. to 39.04 ± 2.95 A.U.; *p* = 0.001), whereas the placebo group showed no significant change. The between-group difference was significant at week 12 (*p* = 0.034). Forearm dermal hydration demonstrated a small increase in the extract group, while a slight decrease was observed in the placebo group, resulting in a statistically significant between-group difference at week 12 (*p* = 0.039). Overall, these findings indicate that oral supplementation with *Centella asiatica* extract was associated with statistically significant increases in dermal hydration, particularly at the cheek site; however, these findings should be interpreted as secondary outcomes rather than confirmatory evidence.

**Figure 4:**
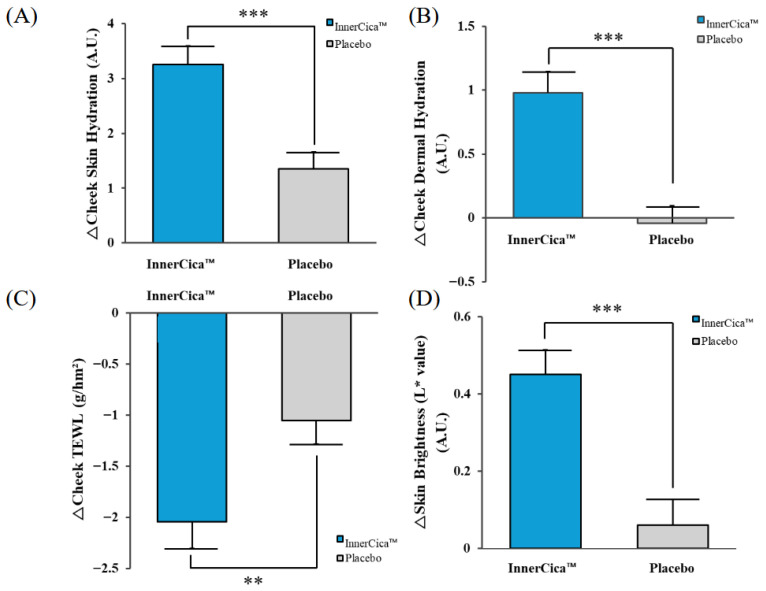
Changes in skin hydration following oral supplementation with *Centella asiatica* extract (InnerCica^™^). Changes in epidermal hydration at the cheek site measured using a Corneometer^®^, and dermal hydration measured using a Moisturemeter^®^ over 12 weeks. The evaluated parameters include (**A**) cheek epidermal hydration, (**B**) cheek dermal hydration, (**C**) cheek transepidermal water loss (TEWL), and (**D**) skin brightness (L* value). Values are presented as mean ± SD (per-protocol analysis). Compared with placebo, the *Centella asiatica* extract group showed significant increases in hydration, particularly at the cheek site. ** *p* < 0.01, *** *p* < 0.001 vs. placebo group.

The temporal changes and between-group differences in epidermal and dermal hydration are visually summarized in Figure 4. As shown, the extract group exhibited a progressive increase in hydration parameters over the 12-week intervention period, particularly at the cheek site, whereas the placebo group showed smaller changes. In contrast, hydration changes at the forearm and dorsal hand sites remained relatively small and showed limited between-group differences.

#### 3.3.3. Effects on Skin Barrier Function

Skin barrier function was assessed by measuring transepidermal water loss (TEWL) using a Tewameter^®^, and the results are presented in Table 4. In the *Centella asiatica* extract group, TEWL at the cheek site decreased significantly over time, from 11.48 ± 2.60 g/hm^2^ at baseline to 9.89 ± 1.96 g/hm^2^ at week 6 and 9.44 ± 1.79 g/hm^2^ at week 12 (*p* < 0.001 and *p* = 0.017 vs. baseline, respectively). This corresponds to an approximate reduction from baseline at week 12. In comparison, the placebo group also showed a reduction from 10.95 ± 3.05 g/hm^2^ to 9.90 ± 2.42 g/hm^2^ over the same period (*p* < 0.001 vs. baseline), corresponding to a smaller decrease. Importantly, the difference in change from baseline was significant at both week 6 (*p* = 0.020) and week 12 (*p* = 0.005).

At the forearm site, TEWL decreased significantly within both groups during the intervention period. In the extract group, TEWL decreased from 7.33 ± 1.73 g/hm^2^ at baseline to 6.40 ± 1.23 g/hm^2^ at week 12 (*p* < 0.001), while the placebo group decreased from 7.15 ± 1.82 g/hm^2^ to 6.34 ± 1.04 g/hm^2^ (*p* < 0.001). However, the between-group difference in change from baseline was not statistically significant at week 12 (*p* = 0.305). Similarly, TEWL measured at the dorsal hand decreased significantly in both groups. In the extract group, values decreased from 10.47 ± 2.31 g/hm^2^ to 8.58 ± 1.50 g/hm^2^ (*p* < 0.001), whereas the placebo group showed a reduction from 9.96 ± 2.50 g/hm^2^ to 8.57 ± 1.76 g/hm^2^ (*p* < 0.001). The between-group difference in change from baseline did not reach statistical significance at week 12 (*p* = 0.063). TEWL showed a statistically significant reduction at the cheek site, whereas changes at the forearm and dorsal hand sites were smaller and not consistently significant. These results indicate that oral supplementation with *Centella asiatica* extract was associated with a greater reduction in TEWL at the cheek site compared with placebo. No significant between-group differences were observed at the forearm and dorsal hand sites, indicating that the effect was site-specific.

#### 3.3.4. Effects on Skin Elasticity

Skin elasticity was evaluated using a Cutometer^®^, and the R2 (Ua/Uf) parameter, representing gross skin elasticity, was used as the primary index (Table 5). In the *Centella asiatica* extract group, skin elasticity showed a statistically significant increase over time, rising from 0.7309 ± 0.0501 E/mm at baseline to 0.7331 ± 0.0509 E/mm at week 6 and 0.7363 ± 0.0496 E/mm at week 12 (*p* = 0.007 and *p* < 0.001 vs. baseline, respectively), although the magnitude of change was small (~0.7% from baseline). In contrast, the placebo group showed no significant change, with values remaining relatively stable (0.7298 ± 0.0464 at baseline and 0.7302 ± 0.0457 at week 12; *p* = 0.783). The between-group comparison demonstrated a statistically significant increase in skin elasticity in the *Centella asiatica* extract group at week 12 (*p* = 0.024); however, the absolute magnitude of change was small (approximately 0.7% from baseline), suggesting a modest improvement rather than a clinically meaningful effect.

#### 3.3.5. Effects on Skin Brightness and Skin Redness

Skin Brightness and redness were evaluated using a spectrophotometer, and the results are summarized in Table 5. In the *Centella asiatica* extract group, skin brightness (L* value) showed a significant increase over time, rising from 63.99 ± 2.12 A.U. at baseline to 64.28 ± 2.06 A.U. at week 6 and 64.44 ± 2.05 A.U. at week 12 (*p* < 0.001 vs. baseline for both time points), although the magnitude of change was small (~0.7% from baseline). In contrast, the placebo group showed no significant change, with values remaining relatively stable (64.11 ± 2.05 A.U. at baseline and 64.17 ± 2.01 A.U. at week 12; *p* = 0.359). The between-group comparison demonstrated a statistically significant increase in L* values in the *Centella asiatica* extract group at both week 6 (*p* = 0.003) and week 12 (*p* < 0.001); however, the magnitude of change was minimal (approximately 0.7% from baseline), indicating a trend toward improvement rather than a clinically meaningful enhancement in skin brightness. For skin redness (a* value), both groups exhibited significant reductions over time. In the extract group, redness decreased from 12.35 ± 1.67 A.U. at baseline to 11.90 ± 1.65 A.U. at week 6 and 11.47 ± 1.52 A.U. at week 12 (*p* < 0.001 vs. baseline for all time points). Similarly, the placebo group showed a decrease from 11.97 ± 1.36 A.U. to 11.36 ± 1.16 A.U. at week 12 (*p* < 0.001 vs. baseline). However, between-group comparisons did not reveal statistically significant differences at either time point (week 6, *p* = 0.336; week 12, *p* = 0.116). These results indicate that oral supplementation with *Centella asiatica* extract was associated with a modest increase in skin brightness, whereas reductions in skin redness were observed in both groups without significant between-group differences.

### 3.4. Safety Outcomes

To further evaluate safety, hematological and biochemical parameters were assessed at baseline and week 12 (Supplementary Table S5). A total of 3 subjects experienced adverse events (1 in the *Centella asiatica* extract group and 2 in the placebo group). All AEs were assessed as unrelated to the investigational product (IP). During the 12-week intervention period, several hematological parameters showed minor statistically significant changes; however, all values remained within normal clinical reference ranges. No notable between-group differences were observed in other safety parameters.

## 4. Discussion

In this randomized, double-blind, placebo-controlled human intervention trial, oral supplementation with *Centella asiatica* extract (200 mg/day) for 12 weeks was associated with statistically significant changes in several skin-related parameters in middle-aged women. Compared with placebo, the extract group exhibited significant reductions in objective wrinkle parameters, including wrinkle depth and volume, accompanied by increases in epidermal and dermal hydration and a reduction in transepidermal water loss (TEWL). Modest changes were also observed in skin elasticity and brightness, although the magnitude of these effects was relatively small. No intervention-related adverse events were identified, and laboratory parameters remained within normal reference ranges during the 12-week intervention period, supporting a favorable safety profile of *Centella asiatica* extract; however, these findings are limited to short-term administration and do not establish long-term safety. These findings provide clinical evidence suggesting that oral *Centella asiatica* extract supplementation may influence certain skin aging-related parameters under controlled study conditions. Importantly, the present results provide confirmatory evidence for the primary endpoint (wrinkle depth reduction), whereas other observed effects should be interpreted as supportive or exploratory.

The observed improvements in skin hydration, elasticity, and wrinkle parameters may be associated with the biological activities of *Centella asiatica*-derived triterpenoids, as reported in previous studies [16,19,33]. However, as only asiaticoside was standardized and quantitatively specified in the present study, the contribution of other triterpenoid constituents cannot be individually determined and should be regarded as indicative rather than conclusive. In addition, while multiple triterpenoid compounds have been reported to contribute to the pharmacological activities of *Centella asiatica*, the present study did not quantify individual non-standardized constituents. Therefore, mechanistic interpretations linking specific compounds to the observed clinical outcomes remain indirect and should be considered hypothesis-generating rather than confirmatory. Recent experimental studies have demonstrated that asiaticoside and madecassoside can stimulate fibroblast proliferation and collagen synthesis, contributing to dermal matrix remodeling [18,39,40]. In addition, triterpenoid constituents have been shown to enhance antioxidant defense systems and mitigate oxidative stress-induced skin damage, as well as modulate inflammatory pathways and support epidermal barrier integrity [18,39,40]. However, molecular mechanisms were not directly evaluated in the present study, and therefore these observations should be interpreted with caution. The clinical findings cannot be directly linked to specific biological pathways.

Skin wrinkle formation is a hallmark of both intrinsic aging and photoaging and is closely associated with collagen degradation and alterations in skin surface topography [4,41,42]. In the present study, three-dimensional skin imaging revealed statistically significant reductions in multiple objective wrinkle parameters, including average wrinkle depth, mean depth of the largest wrinkle, total wrinkle volume, and surface roughness following *Centella asiatica* extract supplementation. These outcomes were derived from objective, instrument-based measurements rather than subjective assessments [34]. The observed reductions in wrinkle depth (approximately 11–14%) fall within the range reported in randomized controlled trials employing comparable objective skin imaging methodologies; however, differences in study design, population characteristics, and intervention modalities should be taken into account when making direct comparisons. Consistent with this, recent randomized controlled trials evaluating oral or topical bioactive compounds—including collagen peptides and other nutraceutical ingredients—have demonstrated improvements in wrinkle-related parameters assessed using validated quantitative techniques [43,44,45,46,47,48].

Improvement in skin hydration represents a clinically relevant finding, given its relationship with skin comfort, elasticity, and barrier integrity [49,50]. The present study demonstrated statistically significant increases in epidermal hydration at the cheek site, together with changes in dermal hydration. However, the magnitude of these changes was relatively small, and their clinical significance should be regarded as indicative rather than definitive. Although the direction of change observed in the present study (i.e., increased skin hydration following supplementation) is generally consistent with previous reports [43,44,45,46,47,48], the magnitude of improvement (approximately 2–7% over a 12-week supplementation period) was more modest compared with the larger increases reported in some prior studies. These differences may be attributable to variations in study design, participant characteristics, baseline skin condition, intervention type, and measurement methodologies. Overall, these findings suggest that oral supplementation may be associated with modest improvements in skin health over a 12-week supplementation period.

Transepidermal water loss (TEWL) is a widely accepted indicator of epidermal barrier function. Elevated TEWL reflects impaired barrier integrity and is associated with increased skin dryness, sensitivity, and accelerated aging [41,49]. In the present study, TEWL was significantly reduced in the *Centella asiatica* extract group at the facial cheek site, whereas no significant between-group differences were observed at other anatomical sites, indicating a site-specific effect. Skin elasticity, assessed using the Cutometer^®^ R2 parameter, was also statistically increased following supplementation. Elastic recovery reflects the biomechanical properties of the skin and is influenced by collagen and elastin networks as well as hydration status [34,51,52]. However, the magnitude of change in elasticity was small, and its clinical relevance should be regarded as indicative rather than definitive. Although previous randomized controlled trials and meta-analyses have reported improvements in skin elasticity following oral or topical interventions, including collagen peptides and other bioactive compounds [43,44,45,46,47,48], the magnitude of change observed in the present study was modest (approximately 0.7%), and direct comparisons across studies remain limited due to differences in study design, intervention type, and measurement methodologies. From a nutritional perspective, the present findings suggest that measurable changes in certain skin parameters can be observed following oral supplementation. Overall, the findings indicate that *Centella asiatica* extract supplementation may influence multiple skin-related parameters; however, the magnitude and consistency of these effects varied across endpoints. Although reductions in skin redness were observed in both groups, no significant between-group differences were detected, suggesting that this effect may not be specifically attributable to the intervention and may reflect external or seasonal influences.

The strengths of this study include its randomized, double-blind, placebo-controlled design, an adequate sample size, and the use of validated, non-invasive, quantitative instruments to assess multiple dimensions of skin health, including wrinkle morphology, hydration, barrier function, elasticity, and skin tone. Nevertheless, several limitations should be acknowledged. First, the study population consisted exclusively of middle-aged women, which may limit the generalizability of the findings to other age groups, sexes, and skin types. Skin physiology and response to nutritional interventions may differ across these groups, and therefore, caution is warranted when extrapolating the findings beyond the study population. Second, only a single dosage of *Centella asiatica* extract was evaluated, precluding assessment of dose–response relationships. The selected dose of 200 mg/day was based on recent clinical and translational evidence, including randomized controlled trials of *Centella asiatica*-derived formulations and comparable nutricosmetic interventions, which have demonstrated efficacy within a similar dose range. In addition, preclinical and pharmacokinetic studies indicate that triterpenoid-rich extracts exhibit dose-dependent biological activity despite inherently low bioavailability, supporting the rationale for a moderate dosing strategy [25,53,54,55,56,57]. Third, a key limitation of this study is the absence of direct mechanistic assessments, such as biomarkers of collagen metabolism, oxidative stress, or epidermal lipid composition. Although multiple objective clinical endpoints were measured, the lack of molecular or biochemical data limits the ability to establish causal relationships between supplementation and the observed clinical outcomes. Consequently, the underlying biological mechanisms remain inferential and should be regarded as indicative rather than definitive. Fourth, the intervention period was limited to 12 weeks; therefore, the findings do not allow conclusions regarding long-term efficacy or safety. Fifth, multiple endpoints were evaluated without formal adjustment for multiplicity, which may increase the likelihood of type I error, particularly for secondary and exploratory outcomes. Future studies incorporating more diverse populations, multiple dosage levels, mechanistic biomarkers, and longer intervention periods are warranted to further elucidate the biological mechanisms and to assess the long-term relevance of *Centella asiatica* supplementation for skin aging.

## 5. Conclusions

This randomized, double-blind, placebo-controlled clinical trial indicates that 12 weeks of supplementation with a standardized *Centella asiatica* extract was associated with changes in several objective skin-related parameters in middle-aged women. Reductions in wrinkle-related parameters and transepidermal water loss were observed, while changes in skin hydration, elasticity, and brightness were modest in magnitude. These effects were more evident at the facial cheek site compared with other anatomical regions. No safety concerns were identified during the 12-week intervention period; however, these findings are limited to short-term administration. Overall, these findings suggest that oral *Centella asiatica* extract supplementation may influence certain skin aging-related parameters under controlled study conditions. While the present findings are based on objective and validated non-invasive measurements, further studies involving diverse populations, extended intervention periods, and the inclusion of mechanistic biomarkers are warranted to elucidate underlying biological mechanisms and to evaluate long-term efficacy and safety.

## Figures and Tables

**Table 1:** Self-evaluated baseline skin parameters of the participants.

Item	Classification	*Centella asiatica* Extract Group (*n* = 56)	Placebo Group (*n* = 56)	*p*-Value ^(2)^
		N (%) ^(1)^		
Skin type	Dry	56 (100.00)	56 (100.00)	1.000
	Normal	0 (0.00)	0 (0.00)	
	Oily	0 (0.00)	0 (0.00)	
	Dry and oily	0 (0.00)	0 (0.00)	
Skin hydration	Sufficient	0 (0.00)	0 (0.00)	0.473
	Normal	9 (16.07)	12 (21.43)	
	Deficient	47 (83.93)	44 (78.57)	
Skin sebum	Glossy	0 (0.00)	0 (0.00)	0.436
	Normal	24 (42.86)	27 (48.21)	
	Deficient	32 (57.14)	29 (51.79)	
Surface roughness	Smooth	4 (7.14)	3 (5.36)	0.908
	Normal	40 (71.43)	43 (76.79)	
	Rough	12 (21.43)	10 (17.86)	
Skin thickness	Thin	19 (33.93)	15 (26.79)	0.108
	Normal	34 (60.71)	41 (73.21)	
	Thick	3 (5.36)	0 (0.00)	
Sensitivity of skin	Yes	1 (1.79)	0 (0.00)	1.000
	No	55 (98.21)	56 (100.00)	

^(1)^ N (Frequency) = Number of answers; % (Percentage) = Number of answers/Total number of subjects (56) × 100. ^(2)^ Analyzed by Chi-square test between the groups.

**Table 2:** Changes in skin wrinkle parameters during the intervention period (PP analysis).

Parameter	Time Point	*Centella asiatica* Extract Group (*n* = 53)	Placebo Group (*n* = 52)	*p*-Value ^(3)^
Average Depth of Wrinkles (μm)	Baseline	53.02 ± 12.06	56.06 ± 11.53	0.190 ^(4)^
	Week 6	49.43 ± 10.20	53.73 ± 11.85	0.028 *
	Change from baseline	−3.58 ± 6.11	−2.33 ± 7.67	0.686
	*p*-value ^(1)^	<0.001	0.019	
	Week 12	47.15 ± 8.29	54.10 ± 11.12	<0.001 ***
	Change from baseline	−5.87 ± 7.82	−1.96 ± 6.09	0.018 *
	*p*-value ^(1)^	<0.001	0.016	
Mean Depth of the Biggest Wrinkle (μm)	Baseline	72.19 ± 22.06	76.06 ± 23.07	0.382 ^(4)^
	Week 6	63.89 ± 17.93	72.81 ± 22.63	0.017 *
	Change from baseline	−8.30 ± 11.28	−3.25 ± 13.93	0.043 *
	*p*-value ^(1)^	<0.001	0.050	
	Week 12	61.83 ± 15.50	73.02 ± 22.41	0.004 **
	Change from baseline	−10.36 ± 16.08	−3.04 ± 12.58	0.011 * ^(4)^
	*p*-value ^(1)^	<0.001	0.084	
Maximum Depth of the Biggest Wrinkle (μm)	Baseline	227.85 ± 91.99	246.08 ± 92.97	0.315 ^(4)^
	Week 6	201.51 ± 72.02	231.25 ± 101.07	0.140
	Change from baseline	−26.34 ± 46.37	−14.83 ± 47.12	0.258
	*p*-value ^(1)^	<0.001	0.016	
	Week 12	197.57 ± 68.21	231.65 ± 91.40	0.023 *
	Change from baseline	−30.28 ± 59.23	−14.42 ± 56.46	0.136
	*p*-value ^(1)^	<0.001	0.037	
Total Wrinkle area (mm^2^)	Baseline	42.35 ± 5.44	41.74 ± 6.10	0.590 ^(4)^
	Week 6	42.14 ± 5.66	41.72 ± 5.79	0.727
	Change from baseline	−0.21 ± 2.97	−0.02 ± 2.49	0.722 ^(4)^
	*p*-value ^(1)^	0.500	0.620	
	Week 12	41.50 ± 5.39	41.43 ± 6.00	0.870
	Change from baseline	−0.85 ± 2.75	−0.32 ± 3.30	0.365 ^(4)^
	*p*-value ^(1)^	0.025	0.521	
Total Wrinkle Volume (mm^3^)	Baseline	2.27 ± 0.70	2.36 ± 0.66	0.478 ^(4)^
	Week 6	2.09 ± 0.58	2.27 ± 0.67	0.082
	Change from baseline	−0.18 ± 0.32	−0.09 ± 0.36	0.338
	*p*-value ^(1)^	<0.001	0.067 ^(2)^	
	Week 12	1.96 ± 0.50	2.26 ± 0.63	0.004 **
	Change from baseline	−0.30 ± 0.38	−0.10 ± 0.33	0.004 **
	*p*-value ^(1)^	<0.001	0.038 ^(2)^	
Total Length of Wrinkles (mm)	Baseline	59.75 ± 8.26	57.75 ± 9.77	0.258 ^(4)^
	Week 6	56.11 ± 8.31	56.67 ± 8.41	0.258
	Change from baseline	−3.61 ± 6.67	−1.08 ± 6.68	0.837
	*p*-value ^(1)^	<0.001 ^(2)^	0.326	
	Week 12	55.70 ± 6.93	55.15 ± 8.40	0.320
	Change from baseline	−4.06 ± 5.26	−2.60 ± 6.55	0.211
	*p*-value ^(1)^	<0.001 ^(2)^	0.007	
Surface Roughness (Ra) (μm)	Baseline	24.60 ± 4.90	25.83 ± 4.79	0.199
	Week 6	22.94 ± 4.39	24.87 ± 5.24	0.027 *
	Change from baseline	−1.66 ± 2.61	−0.96 ± 3.55	0.514
	*p*-value ^(1)^	<0.001	0.021	
	Week 12	22.04 ± 3.54	24.77 ± 4.62	0.011 **
	Change from baseline	−2.57 ± 3.12	−1.06 ± 2.84	0.023 *
	*p*-value ^(1)^	<0.001	0.011	
Maximum Height of Surface Profile (Ry) (μm)	Baseline	436.57 ± 117.28	439.35 ± 109.30	0.900 ^(4)^
	Week 6	409.85 ± 94.95	429.29 ± 120.66	0.549
	Change from baseline	−26.72 ± 69.46	−10.06 ± 75.46	0.154
	*p*-value ^(1)^	0.008	0.059	
	Week 12	396.15 ± 96.37	430.29 ± 106.45	0.076
	Change from baseline	−40.42 ± 81.78	−9.06 ± 72.11	0.086
	*p*-value ^(1)^	0.002	0.366	

Changes from baseline in key wrinkle parameters assessed using a three-dimensional skin imaging system (PRIMOS^®^), including average wrinkle depth, mean depth of the biggest wrinkle, maximum wrinkle depth, total wrinkle area, total wrinkle volume, surface roughness (Ra) and maximum height of surface profile (Ry) after 6 or 12 weeks of intervention. Values are presented as mean ± SD (PP analysis). ^(1)^ Analyzed by Wilcoxon signed rank test compared to baseline within each group. ^(2)^ Analyzed by paired *t*-test compared to baseline within each group. ^(3)^ Analyzed by Mann–Whitney U test for the *Centella asiatica* extract group vs placebo group. ^(4)^ Analyzed by Independent *t*-test for the *Centella asiatica* extract group vs placebo group. * *p* < 0.05, ** *p* < 0.01, *** *p* < 0.001 vs. placebo group.

**Table 3:** Changes in skin hydration during the intervention period (PP analysis).

Parameter	Time Point	*Centella asiatica* Extract Group (*n* = 53)	Placebo Group (*n* = 52)	*p*-Value ^(3)^
Cheek Skin Hydration (A.U.)	Baseline	44.83 ± 4.73	44.10 ± 3.75	0.382 ^(4)^
	Week 6	46.89 ± 4.76	45.10 ± 3.99	0.003 **
	Change from baseline	2.06 ± 1.97	1.00 ± 1.50	0.008 **
	*p*-value ^(1)^	<0.001	<0.001	
	Week 12	48.09 ± 5.08	45.45 ± 4.17	<0.001 ***
	Change from baseline	3.26 ± 2.48	1.35 ± 2.26	<0.001 ***
	*p*-value ^(1)^	<0.001	<0.001	
Forearm Skin Hydration (A.U.)	Baseline	37.88 ± 7.35	35.93 ± 8.52	0.213 ^(4)^
	Week 6	38.19 ± 6.77	36.70 ± 7.88	0.299 ^(4)^
	Change from baseline	0.31 ± 1.79	0.77 ± 2.52	0.276
	*p*-value ^(1)^	0.204 ^(2)^	0.033 ^(2)^	
	Week 12	39.28 ± 6.12	37.53 ± 7.39	0.190 ^(4)^
	Change from baseline	1.40 ± 2.94	1.60 ± 2.82	0.564
	*p*-value ^(1)^	0.001 ^(2)^	<0.001 ^(2)^	
Dorsal Hand Skin Hydration (A.U.)	Baseline	38.27 ± 6.98	37.01 ± 6.65	0.346 ^(4)^
	Week 6	38.76 ± 6.53	37.3 ± 6.83	0.187
	Change from baseline	0.49 ± 1.68	0.33 ± 1.62	0.848
	*p*-value ^(1)^	0.055	0.105	
	Week 12	39.40 ± 6.00	38.10 ± 6.31	0.279
	Change from baseline	1.13 ± 2.31	1.09 ± 2.11	0.626
	*p*-value ^(1)^	0.002	<0.001	
Cheek Dermal Hydration (A.U.)	Baseline	36.10 ± 2.64	36.36 ± 3.23	0.650 ^(4)^
	Week 6	36.89 ± 2.64	36.60 ± 3.19	0.727
	Change from baseline	0.79 ± 1.27	0.24 ± 0.96	0.015 * ^(4)^
	*p*-value ^(1)^	<0.001 ^(2)^	0.082 ^(2)^	
	Week 12	37.08 ± 2.48	36.32 ± 3.27	0.182 ^(4)^
	Change from baseline	0.98 ± 1.20	−0.04 ± 0.96	<0.001 *** ^(4)^
	*p*-value ^(1)^	<0.001 ^(2)^	0.767 ^(2)^	
Forearm Dermal Hydration (A.U.)	Baseline	28.63 ± 3.41	28.82 ± 3.31	0.768 ^(4)^
	Week 6	28.83 ± 3.39	28.88 ± 3.36	0.835
	Change from baseline	0.20 ± 1.00	0.06 ± 1.37	0.331
	*p*-value ^(1)^	0.020	0.771	
	Week 12	28.90 ± 3.12	28.50 ± 3.20	0.455
	Change from baseline	0.27 ± 1.41	−0.32 ± 1.46	0.039 *
	*p*-value ^(1)^	0.112	0.187	
Dorsal Hand Dermal Hydration	Baseline	38.31 ± 3.23	37.15 ± 3.94	0.822 ^(4)^
	Week 6	38.79 ± 3.11	38.18 ± 4.16	0.395 ^(4)^
	Change from baseline	0.48 ± 1.04	0.03 ± 1.32	0.118
	*p*-value ^(1)^	0.001 ^(2)^	0.858	
	Week 12	39.04 ± 2.95	38.20 ± 4.24	0.242 ^(4)^
	Change from baseline	0.73 ± 1.50	0.05 ± 1.61	0.034 *
	*p*-value ^(1)^	0.001 ^(2)^	0.814	

Changes from baseline in epidermal hydration at the cheek, forearm, and dorsal hand sites measured using a Corneometer^®^, and changes from baseline in dermal hydration measured using a Moisturemeter^®^ after 12 weeks of supplementation. Data are presented as mean ± SD (PP analysis). ^(1)^ Analyzed by Wilcoxon signed rank test compared to baseline within each group. ^(2)^ Analyzed by paired *t*-test compared to baseline within each group. ^(3)^ Analyzed by Mann–Whitney U test for the *Centella asiatica* extract group vs placebo group. ^(4)^ Analyzed by Independent *t*-test for the InnerCica^TM^ group vs placebo group. * *p* < 0.05, ** *p* < 0.01, *** *p* < 0.001 vs. placebo group.

**Table 4:** Changes in transepidermal water loss (TEWL) during the intervention period (PP analysis).

Parameter	Time Point	*Centella asiatica* Extract Group (*n* = 53)	Placebo Group (*n* = 52)	*p*-Value ^(2)^
Cheek TEWL (g/hm^2^)	Baseline	11.48 ± 2.60	10.95 ± 3.05	0.337 ^(3)^
	Week 6	9.89 ± 1.96	10.03 ± 2.64	0.485
	Change from baseline	−1.59 ± 1.50	−0.92 ± 1.44	0.020 *
	*p*-value ^(1)^	<0.001	<0.001	
	Week 12	9.44 ± 1.79	9.90 ± 2.42	0.642
	Change from baseline	−2.04 ± 1.91	−1.05 ± 1.66	0.005 **
	*p*-value ^(1)^	0.017	0.341	
Forearm TEWL (g/hm^2^)	Baseline	7.33 ± 1.73	7.15 ± 1.82	0.608 ^(3)^
	Week 6	6.68 ± 1.31	6.51 ± 1.16	0.621
	Change from baseline	−0.65 ± 0.94	−0.64 ± 1.19	0.686
	*p*-value ^(1)^	<0.001	<0.001	
	Week 12	6.40 ± 1.23	6.34 ± 1.04	0.969
	Change from baseline	−0.92 ± 0.96	−0.81 ± 1.35	0.305
	*p*-value ^(1)^	<0.001	<0.001	
Dorsal Hand TEWL (g/hm^2^)	Baseline	10.47 ± 2.31	9.96 ± 2.50	0.282 ^(3)^
	Week 6	9.03 ± 1.61	8.78 ± 1.90	0.245
	Change from baseline	−1.44 ± 1.63	−1.18 ± 1.62	0.503
	*p*-value ^(1)^	<0.001	<0.001	
	Week 12	8.58 ± 1.50	8.57 ± 1.76	0.658
	Change from baseline	−1.89 ± 1.53	−1.39 ± 1.47	0.063
	*p*-value ^(1)^	<0.001	<0.001	

Changes from baseline in transepidermal water loss at the cheek, forearm, and dorsal hand sites measured using a Tewameter^®^ after 12 weeks of intervention. Data are presented as mean ± SD (PP analysis). ^(1)^ Analyzed by Wilcoxon signed rank test compared to baseline within each group. ^(2)^ Analyzed by Mann–Whitney U test for the *Centella asiatica* extract group vs placebo group. ^(3)^ Analyzed by Independent *t*-test for the *Centella asiatica* extract group vs placebo group. * *p* < 0.05, ** *p* < 0.01 vs. placebo group.

**Table 5:** Changes in skin elasticity and skin tone during the intervention period (PP analysis).

Parameter	Time Point	*Centella asiatica* Extract Group (*n* = 53)	Placebo Group (*n* = 52)	*p*-Value ^(3)^
Skin Elasticity (R2 Parameter) (E/mm)	Baseline	0.7309 ± 0.0501	0.7298 ± 0.0464	0.907 ^(4)^
	Week 6	0.7331 ± 0.0509	0.7310 ± 0.0465	0.649
	Change from baseline	0.0023 ± 0.0052	0.0013 ± 0.0054	0.649
	*p*-value ^(1)^	0.007	0.097 ^(2)^	
	Week 12	0.7363 ± 0.0496	0.7302 ± 0.0457	0.307 ***
	Change from baseline	0.0054 ± 0.0093	0.0004 ± 0.0109	0.024 *
	*p*-value ^(1)^	<0.001	0.783 ^(2)^	
Skin Brightness (L* value) (A.U.)	Baseline	63.99 ± 2.12	64.11 ± 2.05	0.768 ^(4)^
	Week 6	64.28 ± 2.06	64.14 ± 2.02	0.730 ^(4)^
	Change from baseline	0.29 ± 0.48	0.04 ± 0.30	0.003 **
	*p*-value ^(1)^	<0.001 ^(2)^	0.405 ^(2)^	
	Week 12	64.44 ± 2.05	64.17 ± 2.01	0.504 ^(4)^
	Change from baseline	0.45 ± 0.46	0.60 ± 0.48	<0.001 ***
	*p*-value ^(1)^	<0.001 ^(2)^	0.359 ^(2)^	
Skin Redness (a* value) (A.U.)	Baseline	12.35 ± 1.67	11.97 ± 1.36	0.205 ^(4)^
	Week 6	11.90 ± 1.65	11.62 ± 1.23	0.337 ^(4)^
	Change from baseline	−0.45 ± 1.52	−0.34 ± 0.58	0.336
	*p*-value ^(1)^	<0.001	<0.001	
	Week 12	11.47 ± 1.52	11.36 ± 1.16	0.663 ^(4)^
	Change from baseline	−0.88 ± 0.70	−0.61 ± 0.56	0.116
	*p*-value ^(1)^	<0.001 ^(2)^	0.011	

Changes from baseline in skin elasticity assessed by the Cutometer^®^ R2 (Ua/Uf) parameter, and changes from baseline in skin brightness (L* value) and skin redness (a* value) measured using a spectrophotometer after 12 weeks of supplementation. Data are presented as mean ± SD (PP analysis). ^(1)^ Analyzed by Wilcoxon signed rank test compared to baseline within each group. ^(2)^ Analyzed by paired *t*-test compared to baseline within each group. ^(3)^ Analyzed by Mann–Whitney U test for the *Centella asiatica* extract group vs placebo group. ^(4)^ Analyzed by Independent *t*-test for the *Centella asiatica* extract group vs placebo group. * *p* < 0.05, ** *p* < 0.01, *** *p* < 0.001 vs. placebo group.

## Data Availability

The data presented in this study are available from the corresponding author upon reasonable request. Due to ethical and privacy considerations, individual participant data is not publicly available.

## Supplementary Materials

The following supporting information can be downloaded at https://www.mdpi.com/article/10.3390/nu18101505/s1. Table S1: Body composition of the participants in the PP population. The body composition was measured at baseline and at Week 12. Table S2: Dietary Intake of Participants during the Intervention Period. Table S3: Self-assessment for lifestyle habits in the PP population. Table S4: Self-assessment of lifestyle habits (ITT population). Table S5: Blood safety test values of the participants in the PP population. Blood test values were measured at the screening visit and in Week 12. Table S6: Measurement sites and instruments for skin evaluation.
