# Supplementary material for: A Randomized, Double-Blind, Placebo-Controlled Trial Assessing the Effects of Oral Centella asiatica Extract on Skin Aging-Related Parameters in Middle-Aged Korean Women
Source: Nutrients. 2026 May 8;18(10):1505. doi: 10.3390/nu18101505 (PMC13209926; doi:10.3390/nu18101505)
Supplement: Supplementary file 1 [file nutrients-18-01505-s001.zip › nutrients-4274701-supplementary.pdf]

Supplementary Table S1. Body composition of the participants in the PP population. The body composition was measured at baseline and at Week 12.

Parameter	Time Point	*Centella asiatica* extract group (N=53)	Placebo group (N=52)	*p*-Value [3]
Weight (kg)	Baseline	58.65 ± 9.23	59.70 ± 7.15	0.515 [4]
	Week 12	58.42 ± 9.28	59.10 ± 7.03	0.593
	Change from baseline	-0.22 ± 1.17	-0.60 ± 1.16	0.158
	p-value [1]	0.043	< 0.001 [2]	
Body fat mass (kg)	Baseline	19.24 ± 6.24	20.23 ± 5.69	0.399 [4]
	Week 12	19.30 ± 6.06	19.78 ± 5.57	0.442
	Change from baseline	0.06 ± 1.93	-0.45 ± 1.30	0.157
	p-value [1]	0.770	0.016 [2]	
Body mass index (kg/m²)	Baseline	23.15 ± 3.33	24.02 ± 3.08	0.165 [4]
	Week 12	23.07 ± 3.35	23.78 ± 2.99	0.190
	Change from baseline	-0.08 ± 0.46	-0.24 ± 0.49	0.154
	p-value [1]	0.075	< 0.001	
Body fat percentage (%)	Baseline	32.20 ± 6.00	33.47 ± 6.60	
	Week 12	32.50 ± 6.03	33.09 ± 6.79	0.305 [4]
	Change from baseline	0.30 ± 3.06	-0.38 ± 1.99	0.639 [4]
	p-value [1]	0.478 [2]	0.176 [2]	0.258
Waist-hip ratio (%)	Baseline	0.84 ± 0.05	0.84 ± 0.44	0.985
	Week 12	0.83 ± 0.05	0.83 ± 0.05	0.377
	Change from baseline	-0.01 ± 0.02	-0.01 ± 0.02	0.644
	p-value [1]	0.003	0.001	

[1] Analyzed by Wilcoxon signed rank test compared to baseline within each group. [2] Analyzed by paired t-test between compared to baseline within each group. [3] Analyzed by Mann-Whitney U test for between the *Centella asiatica* extract group vs placebo group. [4] Analyzed by Independent t-test for the *Centella asiatica* extract group vs placebo group. * $p < 0.05$, ** $p < 0.01$, *** $p < 0.001$ vs. placebo group.

Supplementary Table S2. Dietary Intake of Participants during the Intervention Period.

Parameter	Time Point	*Centella asiatica* extract group (N=53)	Placebo group (N=52)	*p*-Value [3]

Energy (kcal/day)	Baseline	1489.73 ± 309.76	1476.36 ± 314.68	0.827 [4)]
	Week 12	1452.40 ± 327.15	1439.00 ± 322.90	0.939
	Change from baseline	-37.33 ± 191.67	-37.35 ± 176.23	0.999 [4)]
	p-value [1)]	0.237	0.133 [2)]	
Carbohydrate (g/day)	Baseline	221.05 ± 47.88	224.88 ± 51.79	0.694 [4)]
	Week 12	219.47 ± 52.79	219.44 ± 52.37	0.998 [4)]
	Change from baseline	-1.58 ± 34.66	-5.44 ± 37.72	0.587 [4)]
	p-value [1)]	0.741 [2)]	0.303 [2)]	
Lipid (g/day)	Baseline	41.65 ± 13.19	38.68 ± 12.26	0.236 [4)]
	Week 12	38.35 ± 11.41	36.74 ± 11.62	0.753
	Change from baseline	-3.30 ± 9.39	-1.95 ± 10.47	0.487 [4)]
	p-value [1)]	0.016	0.186 [2)]	
Protein (g/day)	Baseline	54.46 ± 14.00	54.67 ± 14.19	0.939 [4)]
	Week 12	54.83 ± 14.26	55.14 ± 14.08	0.798
	Change from baseline	0.37 ± 11.98	0.47 ± 8.81	0.880
	p-value [1)]	0.655	0.700 [2)]	
Grain (servings/day)	Baseline	2.47 ± 0.60	2.57 ± 0.65	0.399 [4)]
	Week 12	2.38 ± 0.64	2.48 ± 0.66	0.471
	Change from baseline	-0.09 ± 0.48	-0.10 ± 0.51	0.987
	p-value [1)]	0.198 [2)]	0.173 [2)]	
Meat/Fish/Eggs/Legumes (servings/day)	Baseline	3.28 ± 1.67	2.74 ± 0.99	0.049 * [4)]
	Week 12	2.91 ± 1.18	2.85 ± 1.09	0.810
	Change from baseline	-0.37 ± 1.33	0.11 ± 0.85	0.113
	p-value [1)]	0.110	0.447	
Vegetables (servings/day)	Baseline	3.63 ± 1.13	3.80 ± 1.47	0.494 [4)]
	Week 12	3.95 ± 1.41	4.18 ± 1.59	0.361
	Change from baseline	0.32 ± 1.55	0.37 ± 1.19	0.857 [4)]
	p-value [1)]	0.125	0.028 [2)]	
Fruits (servings/day)	Baseline	1.02 ± 0.76	1.01 ± 0.73	0.988 [4)]
	Week 12	1.15 ± 1.00	0.92 ± 0.72	0.412

	Change from baseline	0.13 ± 0.94	-0.10 ± 0.72	0.162 [4)]
	p-value [1)]	0.378	0.209	
Milk/Dairy (servings/day)	Baseline	0.53 ± 0.44	0.64 ± 0.58	0.290 [4)]
	Week 12	0.58 ± 0.59	0.51 ± 0.55	0.433
	Change from baseline	0.05 ± 0.56	-0.13 ± 0.44	0.145
	p-value [1)]	0.890	0.048	
Fat/Sugar (servings/day)	Baseline	4.11 ± 2.02	3.78 ± 2.15	0.428 [4)]
	Week 12	4.38 ± 2.37	3.83 ± 1.98	0.198
	Change from baseline	0.27 ± 1.94	0.04 ± 2.32	0.581 [4)]
	p-value [1)]	0.324	0.845	

[1)] Analyzed by Wilcoxon signed rank test compared to baseline within each group. [2)] Analyzed by paired t-test between compared to baseline within each group. [3)] Analyzed by Mann-Whitney U test for between the *Centella asiatica* extract group vs placebo group. [4)] Analyzed by Independent t-test for the *Centella asiatica* extract group vs placebo group. Data are presented as mean ± SD. Dietary intake was assessed using dietary records at baseline and after 12 weeks of supplementation. All variables are expressed with appropriate units: energy (kcal/day), macronutrients (g/day), and food group intake (servings/day).

Supplementary Table S3. Self-assessment for lifestyle habits in the PP population.

Item	Classification	Test group (N=53) Before N	Before %	After 12 weeks N	After 12 weeks %	Control group (N=52) Before N	Before %	After 12 weeks N	After 12 weeks %	*p*-value (Test)	*p*-value (Control)
Q1. Average daily UV exposure	<1 h	17	32.08	13	24.53	12	23.08	15	28.85	0.683	0.791
	1–3 h	33	62.26	37	69.81	35	67.31	32	61.54		
	>3 h	3	5.66	3	5.66	5	9.62	5	9.62		
Q2. Average daily sleep time	≤5 h	3	5.66	3	5.66	1	1.92	2	3.85	0.941	0.750
	5–8 h	45	84.91	46	86.79	49	94.23	47	90.38		
	≥8 h	5	9.43	4	7.55	2	3.85	3	5.77		
Q3. Cosmetics used	Skin	53	100.00	53	100.00	52	100.00	52	100.00	1.000	1.000
	Lotion	53	100.00	53	100.00	52	100.00	52	100.00		
	Essence	0	0.00	0	0.00	0	0.00	0	0.00		
	Cream	53	100.00	53	100.00	52	100.00	52	100.00		
	Sunscreen	53	100.00	53	100.00	52	100.00	52	100.00		
Q4. Cleansing method	Single cleansing	38	71.70	40	75.47	41	78.85	42	80.77	0.826	0.807
	Double cleansing	15	28.30	13	24.53	11	21.15	10	19.23		
Q5. Water intake per	≤3 cups	13	24.53	14	26.42	13	25.00	11	21.15	0.562	0.915

day	4–6 cups	30	56.60	26	49.06	28	53.85	31	59.62		
	7–9 cups	9	19.68	13	24.53	8	15.38	8	15.38		
	≥10 cups	1	1.89	0	0.00	3	5.77	2	3.85		
Q6. Caffeine intake per day	≤1 cup	24	45.28	25	47.17	20	38.46	19	36.54	0.796	0.795
	2–3 cups	24	45.28	24	45.28	23	44.23	26	50.00		
	>3 cups	1	1.89	0	0.00	0	0.00	0	0.00		
	None	4	7.55	4	7.55	9	17.31	7	13.46		
Q7. Smoking status	Non-smoker	53	100.00	53	100.00	52	100.00	52	100.00	1.000	1.000
	≤10 cigarettes	0	0.00	0	0.00	0	0.00	0	0.00		
	>10 cigarettes	0	0.00	0	0.00	0	0.00	0	0.00		
Q8. Alcohol consumption	None	40	75.47	42	79.25	48	92.31	43	82.69	0.891	0.235
	≤1 drink	12	22.64	10	18.87	4	7.69	9	17.31		
	2–3 drinks	1	1.89	1	1.89	0	0.00	0	0.00		

N (Frequency) = Number of answers. % (Percentage) = Number of answers / Total number of subjects × 100. *p*-values were calculated using the Chi-square test.

Supplementary Table S4. Self-assessment of lifestyle habits (ITT population).

Item	Classification	Test group (N=56) Before N	Before %	After 12 weeks N	After 12 weeks %	Control group (N=56) Before N	Before %	After 12 weeks N	After 12 weeks %	*p*-value (Test)	*p*-value (Control)
Q1. Average daily UV exposure	<1 h	19	33.93	15	26.79	15	26.79	18	32.14	0.707	0.817
	1–3 h	34	60.71	38	67.86	36	64.29	33	58.93		
	>3 h	3	5.36	4	5.36	5	8.93	5	8.93		
Q2. Average daily sleep time	≤5 h	3	5.36	3	5.36	1	1.79	2	3.57	0.941	0.773
	5–8 h	48	85.71	49	87.50	52	92.86	50	89.29		
	≥8 h	5	8.93	4	7.14	3	5.36	4	7.14		
Q3. Cosmetics used	Skin	56	100.00	56	100.00	56	100.00	56	100.00	1.000	1.000
	Lotion	56	100.00	56	100.00	56	100.00	56	100.00		
	Essence	0	0.00	0	0.00	0	0.00	0	0.00		
	Cream	56	100.00	56	100.00	56	100.00	56	100.00		
	Sunscreen	56	100.00	56	100.00	56	100.00	56	100.00		
Q4. Cleansing method	Single cleansing	41	73.21	43	76.79	44	78.57	45	80.36	0.828	0.815
	Double cleansing	15	26.79	13	23.21	12	21.43	11	19.64		
Q5. Water intake per day	≤3 cups	13	23.21	14	25.00	14	25.00	12	21.43	0.568	0.921
	4–6 cups	33	58.93	29	51.79	31	55.36	34	60.71		
	7–9 cups	9	16.07	13	23.21	8	14.29	8	14.29		
	≥10 cups	1	1.79	0	0.00	3	5.36	2	3.57		
Q6. Caffeine intake per day	≤1 cup	25	44.64	26	46.43	22	39.29	21	37.50	0.797	0.801
	2–3 cups	25	44.64	25	44.64	25	44.64	28	50.00		
	>3 cups	1	1.79	0	0.00	0	0.00	0	0.00		
	None	5	8.93	5	8.93	9	16.07	7	12.50		
Q7. Smoking status	Non-smoker	56	100.00	56	100.00	56	100.00	56	100.00	1.000	1.000
	≤10 cigarettes	0	0.00	0	0.00	0	0.00	0	0.00		

	>10 cigarettes	0	0.00	0	0.00	0	0.00	0	0.00		
Q8. Alcohol consumption	None	43	76.79	45	80.36	51	91.07	46	82.14	0.893	0.267
	≤1 drink	12	21.43	10	17.86	5	8.93	10	17.86		
	2–3 drinks	1	1.79	1	1.79	0	0.00	0	0.00		

N (Frequency) = Number of answers. % (Percentage) = Number of answers / Total number of subjects × 100. *p*-values were calculated using the Chi-square test.

Supplementary Table S5. Blood safety test values of the participants in the PP population. Blood test values were measured at the screening visit and in Week 12.

Parameter	Time Point	*Centella asiatica* extract group (N=53)	Placebo group (N=52)	*p*-Value [3]
WBC (K/uL)	Baseline	6.09 ± 1.39	5.83 ± 1.49	0.354 [4]
	Week 12	5.54 ± 1.44	5.63 ± 1.27	0.595
	Change from baseline	-0.55 ± 1.15	-0.20 ± 1.10	0.061
	p-value [1]	0.001	0.205 [2]	
RBC (M/uL)	Baseline	4.37 ± 0.26	4.42 ± 0.39	0.393 [4]
	Week 12	4.39 ± 0.26	4.39 ± 0.36	0.971 [4]
	Change from baseline	0.02 ± 0.20	-0.03 ± 0.22	0.192 [4]
	p-value [1]	0.449 [2]	0.284 [2]	
Hemoglobin (g/dL)	Baseline	12.82 ± 1.10	12.96 ± 1.03	0.512 [4]
	Week 12	12.79 ± 1.19	12.90 ± 1.05	0.944
	Change from baseline	-0.03 ± 0.66	-0.06 ± 0.60	0.836 [4]
	p-value [1]	0.793	0.476 [2]	
Hematocrit (%)	Baseline	38.89 ± 2.92	39.52 ± 3.26	0.300 [4]
	Week 12	39.88 ± 3.23	39.88 ± 3.11	0.573
	Change from baseline	0.99 ± 1.88	0.36 ± 2.16	0.116 [4]
	p-value [1]	< 0.001	0.231 [2]	
Neutrophil (%)	Baseline	58.55 ± 9.11	56.48 ± 8.75	0.239 [4]
	Week 12	55.87 ± 7.93	55.38 ± 8.77	0.768 [4]
	Change from baseline	-2.68 ± 8.11	-1.10 ± 8.02	0.334
	p-value [1]	0.020 [2]	0.329 [2]	
Lymphocyte (%)	Baseline	33.62 ± 8.26	35.77 ± 8.56	0.194 [4]
	Week 12	35.91 ± 7.31	36.83 ± 8.27	0.546 [4]

	Change from baseline	2.28 ± 7.67	1.06 ± 7.41	0.482
	p-value [1)]	0.035 [2)]	0.308 [2)]	
Monocyte (%)	Baseline	5.36 ± 1.26	5.56 ± 1.51	0.464 [4)]
	Week 12	5.57 ± 1.50	5.33 ± 1.37	0.377
	Change from baseline	0.21 ± 1.26	-0.23 ± 1.02	0.074
	p-value [1)]	0.280	0.099	
Eosinophil (%)	Baseline	2.08 ± 1.52	1.87 ± 1.33	0.452 [4)]
	Week 12	2.13 ± 1.49	1.92 ± 1.34	0.568
	Change from baseline	0.06 ± 1.08	0.06 ± 1.24	0.867
	p-value [1)]	0.706	0.823	
AST (IU/L)	Baseline	24.43 ± 5.66	26.88 ± 7.31	0.057 [4)]
	Week 12	23.92 ± 6.66	26.04 ± 6.73	0.018 [*]
	Change from baseline	-0.51 ± 5.68	-0.85 ± 5.05	0.657
	p-value [1)]	0.167	0.417	
ALT (IU/L)	Baseline	19.15 ± 8.31	21.71 ± 11.59	0.197 [4)]
	Week 12	17.89 ± 11.26	19.37 ± 8.46	0.039 [*]
	Change from baseline	-1.26 ± 10.09	-2.35 ± 7.61	0.132
	p-value [1)]	0.002	0.074	
γ-GTP (IU/L)	Baseline	18.96 ± 12.32	19.88 ± 11.59	0.694 [4)]
	Week 12	19.26 ± 14.06	19.88 ± 13.59	0.482
	Change from baseline	0.30 ± 7.03	0.00 ± 6.72	0.990
	p-value [1)]	0.733	0.765	
Total Cholesterol (mg/dL)	Baseline	225.02 ± 40.95	217.44 ± 36.23	0.318 [4)]
	Week 12	216.36 ± 34.65	217.77 ± 40.18	0.686
	Change from baseline	-8.66 ± 28.56	0.33 ± 25.23	0.131
	p-value [1)]	0.030	0.926 [2)]	
Triglycerides (mg/dL)	Baseline	108.70 ± 52.45	111.79 ± 55.93	0.771 [4)]
	Week 12	100.68 ± 53.74	118.87 ± 54.05	0.025 [*]
	Change from baseline	-8.02 ± 54.23	7.08 ± 59.13	0.032 [*]
	p-value [1)]	0.088	0.181	

HDL-Cholesterol (mg/dL)	Baseline	69.94 ± 16.19	68.31 ± 11.09	0.548 [4)]
	Week 12	76.11 ± 17.30	71.35 ± 12.83	0.070
	Change from baseline	6.17 ± 10.41	3.04 ± 7.72	0.063
	p-value [1)]	< 0.001	0.007 [2)]	
LDL-Cholesterol (mg/dL)	Baseline	131.64 ± 32.30	128.19 ± 32.36	0.625 [4)]
	Week 12	127.40 ± 35.00	130.31 ± 33.64	0.665 [4)]
	Change from baseline	-4.25 ± 25.00	2.12 ± 23.01	0.467
	p-value [1)]	0.222 [2)]	0.510 [2)]	
Glucose (mg/dL)	Baseline	89.36 ± 11.03	89.46 ± 9.34	0.959 [4)]
	Week 12	89.42 ± 7.76	87.08 ± 6.52	0.298
	Change from baseline	0.06 ± 8.13	-2.38 ± 7.27	0.045 *
	p-value [1)]	0.314	0.051	

[1)] Analyzed by Wilcoxon signed rank test compared to baseline within each group. [2)] Analyzed by paired t-test between compared to baseline within each group. [3)] Analyzed by Mann-Whitney U test for between the *Centella asiatica* extract group vs placebo group. [4)] Analyzed by Independent t-test for the *Centella asiatica* extract group vs placebo group. * $p < 0.05$, ** $p < 0.01$, *** $p < 0.001$ vs. placebo group. Abbreviations: WBC, white blood cell; RBC, red blood cell; AST, aspartate aminotransferase; ALT, alanine aminotransferase; γ-GTP, γ-glutamyl transferase; HDL, high-density lipoprotein; LDL, low-density lipoprotein.

Supplementary Table S6. Measurement sites and instruments for skin evaluation.

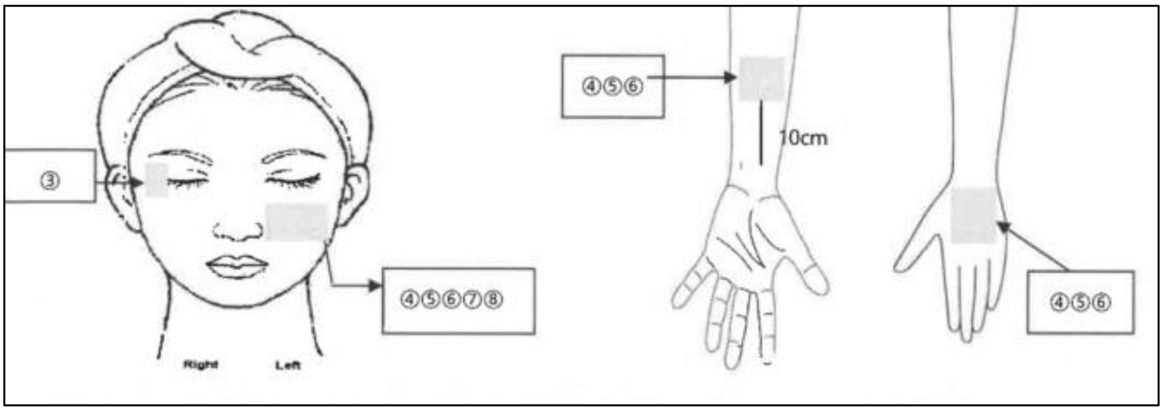

No.	Assessment parameter	Device	Measurement site	Time point
1	Body composition	InBody® 330	Whole body	0 and 12 weeks
2	Clinical photography	VISIA® CR	Frontal and lateral face (L/F/R)	0, 6, and 12 weeks
3	Skin wrinkles	PRIMOS® CR	Periocular region (L or R)	0, 6, and 12 weeks
4	Skin hydration	Corneometer®	Cheek, forearm, hand (L	0, 6, and 12

			or R)	weeks
5	Skin hydration	Moisturemeter® D	Cheek, forearm, hand (L or R)	0, 6, and 12 weeks
6	Transepidermal water loss	Tewameter®	Cheek, forearm, hand (L or R)	0, 6, and 12 weeks
7	Skin elasticity	Cutometer®	Cheek (L or R)	0, 6, and 12 weeks
8	Skin color (brightness and redness)	Spectrophotometer®	Cheek (L or R)	0, 6, and 12 weeks
9	Blood analysis	External clinical laboratory	-	Screening and week 12
10	Pregnancy test	Urine pregnancy test kit	-	Screening and week 12

Schematic illustration of the facial, forearm, and hand skin measurement sites and the dermatological assessment devices used in the clinical trial. Facial measurements were performed on the cheek and periocular regions, while additional measurements were conducted on the forearm and hand. Skin wrinkle analysis was conducted using PRIMOS® CR, hydration using Corneometer® and Moisturmeter® devices, transepidermal water loss using Tewameter®, skin elasticity using Cutometer®, and skin color (brightness and redness) using a spectrophotometer. Whole-body composition was analyzed using InBody® 330. Measurements were conducted at baseline, week 6, and week 12 depending on the parameter. Abbreviations: L, left; R, right. Measurement schedules: 0/6/12 weeks unless otherwise specified.
